# Valorization of Vine Shoot Waste into Phenolic-Rich Liquors for Laccase-Mediated Functionalization of Starch

**DOI:** 10.3390/foods15122177

**Published:** 2026-06-16

**Authors:** Jorge García-Montalvo, Lucía Olmo-García, Nuria Moreno-Rúa, David Oreja-Remartínez, Jorge Fernández-Sánchez, Alegría Carrasco-Pancorbo, Miguel Ladero, Juan M. Bolivar

**Affiliations:** 1 FQPIMA Group, Chemical and Materials Engineering Department, Faculty of Chemical Sciences, Complutense University of Madrid, 28040 Madrid, Spain; jorgar10@ucm.es (J.G.-M.); mladerog@ucm.es (M.L.); 2 Department of Analytical Chemistry, Faculty of Sciences, University of Granada, 18071 Granada, Spainjffernan@ugr.es (J.F.-S.);

**Keywords:** vine shoots, phenolic-rich liquors, citric acid pretreatment, laccase, laccase-mediated functionalization, starch functionalization, phenolic incorporation, antioxidant activity, agro-food waste valorization

## Abstract

Vine shoot residues represent an abundant lignocellulosic by-product of the wine industry and a promising source of phenolic compounds with potential functional applications. In this work, a biocatalytic strategy combining aqueous citric acid treatment and subsequent laccase-mediated oxidation was developed for the valorization of vine shoot-derived phenolic liquors. The pretreatment was optimized by response surface methodology, and the selected conditions, 190 °C, 75 min, and 0.82% citric acid, yielded a pretreated solid containing 2.9 ± 0.02% hemicellulose, 47.5 ± 0.20% cellulose, and 51.8 ± 1.87% lignin, together with a phenolic-rich liquor containing 27.66 ± 0.39 mg GAE g^−1^ dry solid. Chemical characterization by UHPLC-timsTOF-MS revealed a complex mixture of phenolic acids, lignin-derived compounds, carbohydrate derivatives, and secondary metabolites. Laccase-catalyzed oxidation was first used as a reactivity assessment step, showing that the phenolic compounds present in the liquor were susceptible to enzymatic transformation. This treatment decreased the total phenolic content, antioxidant capacity, and antimicrobial activity of the liquor. Subsequently, enzymatic oxidation was carried out in the presence of starch, yielding washed starch solids with retained Folin-reactive phenolic content of approximately 4 mg GAE g^−1^ starch and measurable antioxidant capacity. Overall, this study demonstrates an integrated valorization route in which citric acid-assisted fractionation of vine shoot residues generates phenolic-rich liquors that can be chemically characterized, enzymatically activated, and directly used for starch functionalization, providing a sustainable strategy to convert agro-industrial residues into bio-based functional systems.

## 1. Introduction

The winemaking industry is one of the main agrifood sectors worldwide, with an estimated annual production of 232 million hL in 2025 according to the International Organization of Vine and Wine (OIV). The European Union (EU) is the largest producer, accounting for approximately 140 million hL, with Italy, France and Spain as the leading countries [1]. During wine production, large quantities of residues are generated, approximately 1650 L and 200 kg of residues per 1000 kg of processed grapes. Among these, grape pomace and vine shoots (VSs) are the most abundant solid by-products, second only to winery liquid streams [2]. VSs are produced after grapevine pruning, generating 200 t per km^2^ of vineyard annually [3]. Traditionally, VSs have been used for burning or soil incorporation. Nevertheless, this residue presents high potential for obtaining value-added compounds [4]. Cellulose and hemicellulose are their main components, offering great potential as sources of fermentable sugars [5]. Other notable fractions include lignin, the second most abundant component and a renewable source of phenolic compounds, as well as secondary metabolites, mainly low-molecular-weight phenolics. Consequently, VSs possess significant potential as a source of value-added products [6].

Biomass-derived phenolics have attracted significant interest due to their antioxidants, antimicrobial, antifungal, anti-inflammatory, and anti-aging activities [2]. These properties endow them with high commercial value, particularly in the pharmaceutical, cosmetic, and materials industries. Grapevines synthesize a diverse range of phenolic compounds, which are produced as secondary metabolites to defend against environmental stress and pathogens. The phenolic profile of VS residues includes stilbenes (notably ε-viniferin and resveratrol), flavonoids (such as catechin, vanillin, luteolin, quercetin, and apigenin), and various phenolic acids (including caffeic, gallic, ferulic, *p*-coumaric, and ellagic acids) [7]. The extraction of these compounds has been a primary focus of VS studies, such as the extraction of stilbenes for human health applications [8], their use as enological additives to improve wine organoleptic properties [4] or the obtention of antioxidant liquor to exploit the residue [6,9].

Additionally, fractionation studies have been conducted to extract phenolic compounds while separating cellulose, hemicellulose, and lignin. Notable among these treatments are autohydrolysis and acid extraction. Autohydrolysis consists of treating biomass with hot water, which disrupts the lignocellulosic matrix. At elevated temperatures, the autoionization of water promotes the hydrolysis of acetyl groups from polysaccharides, releasing organic acids such as acetic acid [10]. The use of an acid catalyst boosts this process, enabling higher yields under milder conditions. These treatments generate complex liquors containing phenolic compounds, sugars, oligosaccharides, and other secondary metabolites [10,11,12], alongside degradation products. Other fractionation methods include organosolv for solubilizing lignin and low-molecular-weight phenolics [5,13,14], deep eutectic solvent (DES) extraction [3], steam explosion [15], and microwave-assisted extraction [5,16,17], among others. However, the complex mixture of phenolics, degradation products, and other metabolites hinders their direct industrial application [10].

A green alternative to valorize these liquors is the enzymatic upgrading of phenolics to detoxify the extracts, polymerize the compounds [18] or produce functional materials via grafting reactions [19]. These reactions are catalyzed by laccase, a versatile oxidative enzyme that reduces oxygen to produce phenoxy radicals. These radicals then undergo coupling, covalent bond cleavage, aromatic ring cleavage [20] or covalent bond formation with polymeric materials like cellulose, chitosan, pectin, and starch [19]. While there is abundant literature on grafting phenolic model compounds [21,22,23], there is limited work on grafting phenolic liquors derived directly from biomass [24,25] and none related to VSs.

Previous studies on vine shoots have mainly focused on the extraction of bioactive compounds, particularly stilbenes and phenolic acids, or on fractionation strategies aimed at recovering fermentable sugars, lignin, cellulose, or oligosaccharides. These works demonstrate the potential of vine shoots as biorefinery feedstock, but the recovered liquors are generally evaluated as antioxidant or antimicrobial extracts rather than used directly as reactive streams for polymer functionalization. In parallel, laccase-mediated oxidation has been applied to purified phenolic compounds and to some complex phenolic residues, such as wine lees extracts, to generate oxidized products or polymeric structures. Phenolic grafting onto polysaccharides has also been reported for model compounds such as ferulic acid, quercetin, or phenolic acids, usually using purified substrates and well-defined polymer matrices. However, the direct use of complex vine shoot-derived phenolic liquors for laccase-mediated starch functionalization remains largely unexplored. The present work addresses this gap by linking citric acid-assisted extraction, enzymatic activation of a complex biomass-derived liquor, and in situ phenolic incorporation into starch in a single valorization route.

In this context, the present work explores the valorization of vine shoot residues through the production of phenolic-rich liquors and their subsequent enzymatic upgrading. A citric acid-catalyzed pretreatment was optimized to promote hemicellulose solubilization and phenolic release, generating complex liquors containing lignin-derived phenolics, carbohydrate derivatives, and secondary metabolites. The reactivity of these liquors toward laccase-catalyzed oxidation was then assessed to determine their suitability as substrates for enzymatic coupling reactions. Finally, the enzymatic treatment was carried out in the presence of starch to evaluate the direct in situ incorporation of liquor-derived phenolic compounds into a polysaccharide matrix. The novelty of this approach lies in integrating biomass fractionation, detailed liquor characterization, enzymatic activation of a complex phenolic stream, and direct polysaccharide functionalization without prior purification of individual phenolic compounds. This approach differs from conventional grafting strategies based on purified phenolic compounds, as it uses a complex biomass-derived liquor directly, linking sustainable extraction, biocatalysis, and agro-industrial residue valorization.

## 2. Materials and Methods

### 2.1. Materials

Vine shoots (VSs) from Vitis vinifera (2021 harvest) were provided by Pago de Carraovejas (Valladolid, Spain). This residue was dried at 40 °C for 8 h in an oven and milled using a blade mill to obtain a particle size below 1 mm. Before each extraction process, the moisture of the residues was measured employing a moisture analyzer Kern MLB 50-3N (Kern, Frankfurt am Main, Germany), ramping up the temperature to 105 °C.

*Escherichia coli* CECT 434 and *Staphylococcus aureus* CECT 435 strains were obtained from the Spanish Type Culture Collection (CECT, Paterna, Spain).

Citric acid, sulfuric acid, formic acid, acetonitrile, and Folin–Ciocalteu reagent were purchased from Scharlau (Barcelona, Spain). Cationic starch, Amylofax^®^ HS A, was purchased from Avebe (Veendam, The Netherlands). Acetic acid, glycerol, and LB medium were obtained from Labkem (Mataró, Spain). 2,4,6-tri(2-pyridyl)-s-triazine (TPTZ), Trolox, iron(II) sulfate heptahydrate, cellobiose, glucose, xylose, rhamnose, and arabinose were purchased from Sigma-Aldrich (Saint Louis, MO, USA), and 2,2-diphenyl-1-picrylhydrazyl was purchased from Alfa Aesar (Haverhill, MA, USA). Laccase from *Myceliophthora thermophila* (Novozym 51003^®^) was kindly provided by Novozymes (Bagsværd, Denmark).

### 2.2. Acidic Pretreatment

Dried and milled VSs were added to a custom-built 316 stainless steel pressure reactor designed and manufactured by the mechanical workshop at Universidad Complutense de Madrid (UCM). The equipment consists of a cylindrical vessel with an internal height of 6.5 cm, an internal diameter of 4.4 cm and a wall thickness of 2 cm, yielding a working volume of 50 mL. The reactor outlet was equipped with a ball valve coupled to a Bourdon tube pressure gauge (Wika, Klingenberg am Main, Germany). The system was equipped with an electric heating resistance, an agitation plate IKA RCT basic (IKA-Werke, Staufen im Breisgau, Germany) and an OMRON E5CN PID (Kyoto, Japan) temperature controller coupled to a thermocouple Pt 1000. A solid/liquid ratio of 7.5% (*w*/*v*) was employed. Citric acid was used as a catalyst for hemicellulose extraction. The experimental conditions of temperature and isothermal time were different in each experiment. Once the reaction medium was prepared, the reactor was heated for 40 min to reach the desired temperature. After reaching the target temperature, the reaction time was initiated. Once the reaction was finished, the reactors were rapidly cooled to room temperature using cold water. Then the reaction medium was filtered by vacuum filtration, the permeate was stored at −20 °C for subsequent analysis and the solid was washed twice with the reaction volume of water. The cleaned solid was dried at 50 °C for 16 h.

### 2.3. Experimental Design

Response surface methodology (RSM) was implemented to optimize the hemicellulose extraction process. Specifically, a Box–Behnken design (BBD) was utilized, incorporating central points to evaluate experimental error, guaranteeing reproducibility, and enhancing statistical reliability. The design assessed three independent variables—temperature, isothermal time, and catalyst concentration—across three levels, resulting in a total of 15 experimental runs.

The dependent variables monitored as responses were the recovered fractions of hemicellulose, cellulose, and lignin, alongside the total phenolic content. The acquired experimental data were evaluated using an analysis of variance (ANOVA) to establish the significance of the models. Furthermore, the adequacy of the model fit and the specific influence of each variable on the responses were verified through the coefficient of determination (R^2^), the adjusted R^2^, and the lack-of-fit test. All experimental design generation, statistical modeling, and optimization procedures were executed using Statgraphics Centurion v19 software (Statgraphics Technologies, Inc., The Plains, VA, USA).

### 2.4. Oxidation Experiments

The laccase reaction was carried out in 22 mL glass vials with a working volume of 5 mL. The vials were incubated at 30 °C under agitation at 300 rpm. The hemicellulose-derived phenolic liquor was diluted 1:1 with phosphate buffer (100 mM, pH 7.0). Subsequently, 100 µL of *Myceliophthora thermophila* laccase (MtL, Novozym 51003; 0.3 g L^−1^, 97.1 U mL^−1^) was added to the reaction medium, corresponding to a final activity of 1.94 U mL^−1^. MtL activity was previously determined from the initial oxygen consumption rate using 1 mM catechol dissolved in 50 mM sodium phosphate buffer at pH 7.0. One unit (U) of enzymatic activity was defined as the amount of enzyme that consumes 1 µmol of oxygen per minute under the assay conditions.

For oxygen-monitoring experiments, dissolved oxygen was continuously measured using an optochemical oxygen sensor equipped with a commercial fiber-optic oxygen probe (FireSting^®^-O2, PyroScience, Aachen, Germany). Oxygen consumption was monitored during the initial oxidation stage using an air flow of 50 mL min^−1^ to evaluate the oxidative activity of MtL toward the phenolic liquor. Under these conditions, oxygen consumption was monitored for 1 h, until no further significant oxygen consumption associated with enzymatic oxidation was detected. For endpoint oxidation experiments, the reaction mixtures were incubated for 24 h at 30 °C under agitation. This longer incubation was selected to ensure completion of the oxidative treatment and to avoid time limitation during the subsequent evaluation of liquor transformation and starch functionalization. The laccase treatment was not designed as an independent optimization step, but as a reactivity assessment and activation step to evaluate whether the phenolic-rich liquor contained compounds susceptible to enzymatic oxidation and subsequent incorporation into starch. Therefore, a single reaction condition was selected using excess reaction time and enzyme loading to avoid enzyme or time limitation during this proof-of-concept study.

Control experiments were performed under the same conditions using phenolic liquor without enzyme and phenolic liquor incubated with heat-inactivated laccase. For deactivation, laccase diluted in phosphate buffer 50 mM, pH 7.0, was incubated at 96 °C for 20 min, although less than 10% of the initial laccase activity was detected according to the catechol oxygen consumption assay. These controls were used to assess non-enzymatic oxidation, time-dependent degradation, and possible effects associated with enzyme preparation. The reaction mixtures were then stored at −20 °C until further analysis.

For the starch functionalization assay, 250 mg of starch was added to the reaction medium before enzyme addition, following the same protocol. Upon completion of the reaction, the mixture was centrifuged at 13,000 rpm for 5 min. The supernatant was recovered, and the remaining solid was washed three times with phosphate buffer (50 mM, pH 7.0), with centrifugation after each cycle. The washed precipitate was frozen at −80 °C and subsequently lyophilized in a Virtis Pro Benchtop freeze dryer (SP Scientific, Stone Ridge, NY, USA) at −80 °C and 0.1 mbar for 16 h. Once dried, the solid was stored in a desiccator until further analysis. Additional controls were included to evaluate non-enzymatic phenolic adsorption and the specific contribution of active laccase: starch incubated with phenolic liquor without enzyme, starch incubated with laccase without phenolic liquor, and starch incubated with phenolic liquor and heat-inactivated laccase. All controls were treated, centrifuged, washed, lyophilized, and analyzed using the same protocol as the laccase-treated samples.

### 2.5. Compositional Analysis

The carbohydrate composition was determined following the National Renewable Energy Laboratory (NREL) protocol NREL/TP-510-42618 with minor modifications [1]. Sugar analysis was performed using a Jasco 2000 series HPLC system equipped with a refractive index detector (RID) maintained at 55 °C. The separation of cellobiose, galacturonic acid, glucose, xylose-galactose-mannose, rhamnose, arabinose, acetic acid, and formic acid was performed on a BP 800-H column (Benson Polymeric Inc. Reno, NV, USA), using H_2_SO_4_ 5 mM as the mobile phase flowing at 0.5 mL/min and 60 °C. Standard calibration curves were established for all analytes across a concentration range of 0–5 g L^−1^.

### 2.6. UHPLC-timsTOF-MS Analysis

Untargeted metabolomic profiling was conducted using ultra-high-performance liquid chromatography coupled to trapped ion mobility time-of-flight mass spectrometry (UHPLC-timsTOF-MS) on an Elute series UHPLC system coupled to a timsTOF Pro 2 mass spectrometer (Bruker Daltonics, Bremen, Germany). Separation was achieved on a Zorbax Eclipse Plus C18 column (4.6 × 150 mm, 1.8 μm; Agilent Technologies, Waldbronn, Germany), maintained at 25 °C. The mobile phases consisted of water with 0.5% acetic acid (*v*/*v*) (A) and acetonitrile (B). A flow rate of 0.8 mL min^−1^ was applied with the following gradient: 0–16 min, 5–60% B; 16–17 min, 60–99% B; 17–18.5 min, 99% B; followed by a total re-equilibration time of 2.5 min. The injection volume was 10 μL.

IMS-MS data acquisition was performed in Full Scan (*m*/*z* 20–1300) and the Parallel Accumulation Serial Fragmentation (PASEF) modes. The ion source operated in negative electrospray ionization mode with a capillary voltage of 4500 V. Auxiliary gases were adjusted as follows: the nebulizer pressure was set to 4.0 bar; the drying gas flow and temperature were set at 8.0 L min^−1^ and 200 °C, respectively; and the sheath gas flow and temperature were set at 4.0 L·min^−1^ and 450 °C, respectively. The TIMS unit employed nitrogen as the drift gas, scanning an ion mobility range from 0.10 to 1.50 V·s/cm^2^. Both the accumulation and ramp times were set to 100 ms.

To ensure mass and mobility accuracy, the system underwent external and internal calibration using sodium formate and Agilent ESI-L Low Concentration Tuning Mix. Raw data were processed using MetaboScape 2023 (Bruker Daltonics, Bremen, Germany), which incorporates the T-Rex 4D algorithm for automated recalibration, peak alignment, and molecular feature selection. Feature extraction criteria included an intensity threshold of 10,000 counts and a minimum 4D peak size of 100 points.

### 2.7. HPLC-DAD

High-performance liquid chromatography coupled with diode-array detection was used for the evaluation of phenolic compounds after enzymatic treatment. A HPLC series 4000 from Jasco Inc. (Tokyo, Japan) was employed. Separation was achieved on a C18/PFP Evosphere column (4.6 × 250 mm, 0.5 µm; Fortis, Cheshire, UK). A gradient between phase A (formic acid 0.5% (*v*/*v*) in water) and phase B (acetonitrile) was applied as follows: 0–40 min, 2–60% B; 40–50 min, 60–98% B; 50–55 min, 98% B; and 55–60 min, 98–2% B. Absorbance at 280 nm was measured.

### 2.8. Total Phenolic Content Measurement

Total phenolic content (TPC) was determined using the Folin–Ciocalteu method according to Ribeiro et al. [26] with small modifications [27]; results were expressed as mg gallic acid equivalents per gram dry solid (mg GAE g^−1^ dry solid, DS) [13,14]. Briefly, 30 µL of sample or 30 mg of starch was mixed with 2.52 mL of Folin–Ciocalteu reagent 5.95% (*v*/*v*) in Milli-Q water. After 5 min, 450 µL of Na_2_CO_3_ 15% (*w*/*v*) was added. Following 2 h of incubation, the absorbance at 765 nm was measured using a UV-1800 spectrophotometer (Shimadzu, Kyoto, Japan). Native starch was employed as a blank control to correct the absorbance. Concentrations were determined by interpolation from a gallic acid standard curve ranging from 0 to 750 mg L^−1^. The results are expressed in mg GAE g^−1^ DS. Since the Folin–Ciocalteu assay is based on reducing capacity rather than phenolic-specific detection, the results should be interpreted as Folin-reactive phenolic equivalents expressed as gallic acid equivalents, rather than as absolute concentrations of individual phenolic compounds.

### 2.9. Antioxidant Capacity

Antioxidant capacity was measured using the DPPH radical scavenging and Ferric Reducing Antioxidant Power (FRAP) assays. In both cases, results were expressed as Trolox equivalent antioxidant capacity (TEAC) per mL or gram DS. Trolox calibration curves were established in ethanol 70% (*v*/*v*) over a concentration range of 0–0.25 g L^−1^.

The DPPH assay was carried out as described by Ozturk et al. [28]. Briefly, 200 µL of sample or 30 mg of starch was mixed with 3.8 mL of 0.1 mM DPPH solution in ethanol and incubated for 1 h. The absorbance was subsequently measured at 516 nm using a Shimadzu UV-1800 spectrophotometer. Native starch was employed as a blank control to correct the absorbance.

The FRAP assay was conducted according to Benzie and Strain [29]. Briefly, FRAP reagent was prepared by mixing 50 mL of acetate buffer (pH 3.6), 5 mL of TPTZ 10 mM in HCl 40 mM, and 5 mL of FeCl_3_·6H_2_O. This reagent was pre-warmed at 37 °C for 30 min. Afterwards, 50 µL of the sample or 30 mg of starch was mixed with 1.5 mL of the FRAP reagent. Finally, the mixture was incubated at 37 °C for 30 min, and the absorbance was measured at 593 nm. Native starch was employed as a blank control to correct the absorbance.

### 2.10. Antimicrobial Properties

The antimicrobial properties of the optimized liquor were evaluated against two bacterial strains: the Gram-negative *Escherichia coli* (CECT 434) and the Gram-positive *Staphylococcus aureus* (CECT 435).

The strain, stored at −80 °C in glycerol 20% (*w*/*v*), was inoculated into 5 mL of LB liquid medium. After 18 h, the bacterial culture was re-inoculated into fresh medium and grown until an optical density at 600 nm (OD_600_) of 0.6 was reached. Subsequently, 1 mL of 5-fold concentrated LB medium was mixed with 4 mL of phenolic liquor at different concentrations. Deionized water (4 mL) was used as a control. Following a 24 h incubation, the OD_600nm_ was measured, and the growth inhibition was calculated according to Equation (1). For each assay, the final volume was 5 mL, and the final LB concentration was kept constant at 1× by mixing 1 mL of 5× LB medium with 4 mL of liquid phase. The liquid phase contained phenolic liquor diluted with sterile deionized water to the desired concentration. For example, 2.0, 1.0, or 0.5 mL of liquor was mixed with 2.0, 3.0, or 3.5 mL of sterile water, respectively. Liquor-only blanks without bacterial inoculum were incubated under the same conditions and used to correct OD_600_ values.

(1) $$Growth\;inhibition(\%)=\left(1-\frac{{OD}_{600}\left(Sample\right)}{{OD}_{600}\left(Control\right)}\right)\times 100$$

## 3. Results and Discussion

### 3.1. Optimization of the Acidic Pretreatment for Hemicellulose Extraction

The acidic pretreatment of vine shoots was optimized by response surface methodology in order to maximize hemicellulose solubilization while enriching the solid fraction in cellulose and lignin. The initial biomass composition was 32.28% of cellulose, 21.92% of hemicellulose (3.61% corresponds to acetyl groups) and 21.92% of lignin.

Unless otherwise stated, cellulose, hemicellulose, and lignin contents are expressed as percentages of the corresponding dry solid fraction analyzed. Therefore, the increase in lignin content from 21.92% in the untreated biomass to 51.8% in the optimized pretreated solid reflects lignin enrichment in the recovered solid after hemicellulose and extractive removal, not an increase in absolute lignin mass. The experimental design matrix and the corresponding responses are summarized in Table 1.

Table 1 shows that hemicellulose content in the pretreated solids decreased markedly under the evaluated conditions, whereas cellulose and lignin contents increased as a result of hemicellulose solubilization and removal of extractives (15% of the initial biomass). Consequently, the corresponding liquid fractions contained solubilized hemicellulose-derived compounds together with extractive-derived components. The most severe extraction conditions, corresponding to experiments 2, 4, 6, and 8, led to the lowest residual hemicellulose contents, all below 4%, together with enrichment in cellulose and lignin. In contrast, experiments performed at the lowest temperature retained hemicellulose contents above 18%, indicating limited fractionation under mild conditions.

These results indicate that temperature was the main factor governing hemicellulose extraction, whereas citric acid concentration exerted a secondary but still relevant effect. In general, high temperatures promoted the disruption of the lignocellulosic matrix and favored hemicellulose solubilization, while the presence of citric acid further enhanced this effect, particularly under the most severe conditions.

To quantitatively evaluate the influence of the studied variables, the responses were fitted to quadratic models. After removal of non-significant terms, simplified models were obtained for hemicellulose, cellulose, and lignin contents, with coefficients of determination (R^2^) above 0.87 in all cases (Equations (2)–(4)).

H (%) = 54.3789 − 0.2595·T − 3.3725·c                                                     R^2^ = 0.92 (2)

C (%) = 11.5622 + 0.145583·T + 0.251976·t + 2.4075·c − 0.0020998·t^2^     R^2^ = 0.87 (3)

L (%) = 8.01625 + 0.216375·T + 5.98071·c − 5.98071·c^2^                            R^2^ = 0.94 (4)

The simplified models confirmed that temperature and catalyst concentration significantly affected all three responses, whereas reaction time only showed a significant effect on cellulose content. Hemicellulose content decreased linearly with increasing temperature and catalyst concentration, while cellulose and lignin enrichment exhibited more complex trends, reflecting the combined effects of hemicellulose solubilization and the relative enrichment or partial solubilization of the remaining biomass fractions.

**Figure 1:**
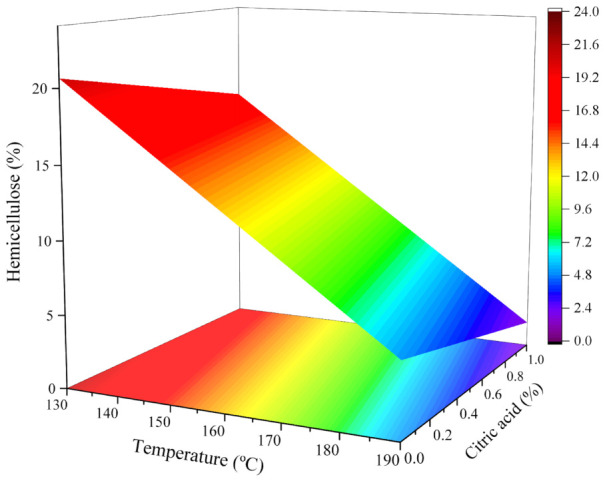
Response surface plot of residual hemicellulose content (%) in the pretreated solid as a function of temperature and citric acid concentration during acid pretreatment of vine shoots.

The response surface plot for hemicellulose content is presented in Figure 1.

As shown in Figure 1, temperature had the strongest effect on hemicellulose removal. Increasing temperature led to a progressive decrease in residual hemicellulose, whereas citric acid further intensified this effect. The lowest hemicellulose contents were predicted at the highest temperature and catalyst concentration tested, indicating that both variables contributed to hemicellulose solubilization. This behavior is consistent with the enhanced hydrolysis of hemicellulosic linkages under acidic hydrothermal conditions as previously described [3,12].

**Figure 2:**
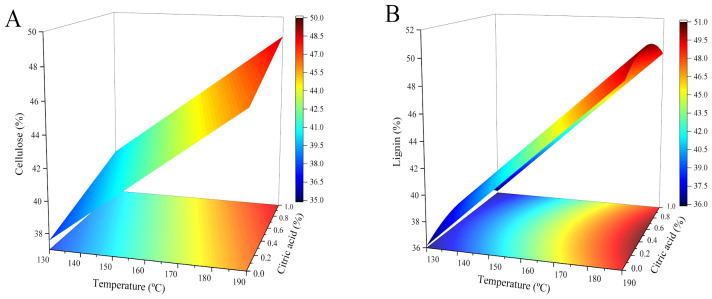
Response surface plots of (**A**) cellulose content (%) and (**B**) lignin content (%) in the pretreated solid as a function of temperature and citric acid concentration during acid pretreatment of vine shoots.

The effects of the process variables on cellulose and lignin enrichment are presented in Figure 2.

Cellulose content increased as hemicellulose was progressively removed from the biomass. This enrichment was mainly driven by temperature, although catalyst concentration also contributed positively. A similar trend was observed for lignin content (Figure 2B), which increased as the carbohydrate fraction was solubilized. However, lignin showed a more complex dependence on catalyst concentration, with maximum lignin content observed at approximately 0.5% citric acid. This trend suggests that, although increasing catalyst concentration favors hemicellulose extraction, higher acid concentrations may also promote partial lignin solubilization, probably through the formation of lower-molecular-weight fragments. These results are in agreement with those from García-Sánchez et al., who also reported higher cellulose and lignin contents at elevated temperatures [30].

A multi-objective optimization was performed to minimize residual hemicellulose while maximizing cellulose and lignin contents in the pretreated solid. The optimal conditions were predicted to be 190 °C, 75 min, and 0.82% citric acid. Under these conditions, the model predicted, with a 95% confidence interval, a solid composition of 1.05% (0–3.32%) hemicellulose with a root mean square error of prediction of 2.23%, 48.31% (46.44–49.91%) cellulose with a root mean square error of prediction of 1.87%, and 50.00% (49.30–52.04%) lignin with a root mean square error of prediction of 1.54%. Experimental validation yielded 2.9 ± 0.02% hemicellulose, 47.5 ± 0.20% cellulose, and 51.8 ± 1.87% total lignin with a solid recovery yield of 49.8% (*w*/*w*) from initial biomass. These values were consistent with the expected trend predicted by the model and fell within or close to the prediction intervals, supporting the usefulness of the model for identifying high-severity conditions that strongly reduce residual hemicellulose. However, the relatively broad prediction interval for hemicellulose indicates limited precision when predicting very low residual hemicellulose values. Overall, the pretreatment efficiently fractionated vine shoots into a cellulose- and lignin-enriched solid and a liquor containing solubilized hemicellulose and phenolic compounds, which was further characterized.

The optimized pretreatment conditions compare favorably with previous reports on vine shoot fractionation. Dávila et al. [12] reported residual hemicellulose contents of around 2.5% after aqueous processing at 205 °C, whereas Rubira et al. [3] reported residual hemicellulose contents of approximately 6.5% under hydrothermal conditions at 205 °C. In the present work, the use of citric acid enabled comparable hemicellulose removal at a lower temperature of 190 °C, while simultaneously producing a liquor enriched in phenolic compounds. These results support the suitability of citric acid-assisted pretreatment as an efficient route for vine shoot fractionation and liquor generation. The selected condition was chosen as a compromise within an integrated valorization strategy. The primary objective of the pretreatment was to obtain a highly fractionated solid with low residual hemicellulose while simultaneously generating a liquor enriched in Folin-reactive phenolic compounds. Therefore, the final condition was not selected solely to maximize TPC, but to balance solid fractionation and liquor valorization. This is relevant because the process was designed as a biorefinery route in which both the pretreated solid and the phenolic-rich liquor are valorized.

### 3.2. Phenolic-Rich Liquor Characterization

**Figure 3:**
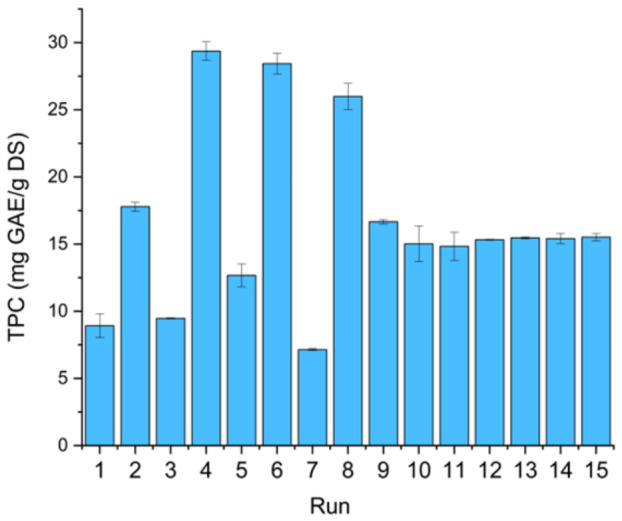
Total phenolic content of phenolic liquors obtained in each run of RSM.

The liquors obtained under the different experimental conditions were first evaluated in terms of total phenolic content (TPC). The results are summarized in Figure 3.

The highest TPC values were obtained for conditions 4, 6, and 8, all performed at 190 °C and isothermal times above 60 min. Under these conditions, TPC values above 25 mg GAE g^−1^ DS were reached. In contrast, experiments carried out at 130 °C (runs 1, 3, 5, and 7) yielded significantly lower TPC values, indicating limited phenolic extraction at low temperatures. These results confirm that temperature is the main factor governing the release of phenolic compounds during the pretreatment, in agreement with the trends observed for hemicellulose solubilization. Increasing temperature enhances the disruption of the lignocellulosic matrix, facilitating the release of both structural phenolics and low-molecular-weight compounds.

To evaluate the possible contribution of non-phenolic compounds to the Folin–Ciocalteu response, selected compounds detected in the liquor or expected from acid pretreatment were tested individually, including furfural, 5-HMF, glucose, xylose, and citric acid. Under the assay conditions used, these compounds showed negligible or non-significant contributions to the TPC signal. Therefore, although the Folin–Ciocalteu assay is not phenolic-specific, the main response observed for the liquor was attributed primarily to Folin-reactive phenolic compounds.

To quantitatively describe this behavior, the experimental data were fitted to a quadratic model, resulting in Equation (5) after removal of non-significant terms

PC = 3.67836 + 0.0802657·T − 0.490107·t + 0.00306317·T·t      R^2^ = 0.86 (5)

**Figure 4:**
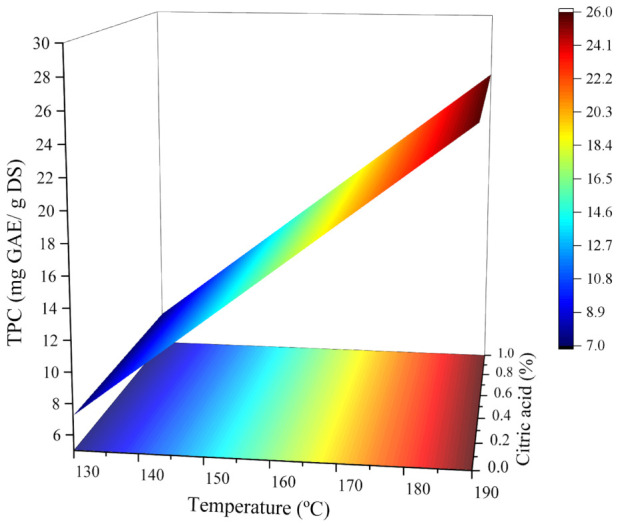
RSM plot of total phenolic content from acid treatment of vine shoots (VSs).

The corresponding response surface is presented in Figure 4. TPC shows a positive and mainly linear dependence on temperature. This behavior suggests that the phenolics predominantly extracted under these conditions are those bound to the lignocellulosic matrix or trapped intracellularly. The contribution of free phenolics is less significant since these compounds exhibit low water solubility. The model predicts a maximum TPC of 25.79 ± 2.93 mg GAE g^−1^ DS under the optimal conditions. Experimental validation yielded 27.66 ± 0.39 mg GAE g^−1^ DS, in agreement with the predicted range and supporting the suitability of the model to identify conditions favoring phenolic release.

The TPC obtained under optimized conditions was comparable to or higher than values reported for vine shoot liquors obtained by related acid or hydrothermal treatments. For example, 22.5 mg GAE g^−1^ DS were reported [10]. Therefore, citric acid-assisted pretreatment allowed efficient phenolic recovery under comparatively mild conditions.

These results are also in agreement with other optimization studies from agro-food residues like the one obtained from *Prunus avium* by García-Sánchez et al. [30] that reaches 32.58 mg GAE g^−1^ DS at 195 °C, 45 min and a liquid/solid ratio (L/S (*w*/*w*)) of 9.

The metabolomic profile of the liquor obtained under optimized conditions was analyzed by ultra-high-performance liquid chromatography coupled to trapped ion mobility time-of-flight mass spectrometry (UHPLC-timsTOF-MS). This multidimensional approach supported tentative annotation by combining accurate mass, fragmentation patterns, and collisional cross section (CCS) values as complementary identification parameters. Untargeted analysis revealed a highly complex composition; therefore, the discussion focuses on the twenty compounds with the highest relative signal intensities (Table 2). An extended list of 60 additional tentatively annotated compounds is provided in Table S1.

The liquor contained a complex mixture of organic acids, carbohydrate-derived compounds, lignin-derived phenolics, and secondary metabolites. The most intense signal corresponded to citric acid, which remained in the reaction medium, indicating limited degradation under optimal conditions. Its main derivatives included citramalic acid and citraconic acid, which stem from the thermal dehydration and decarboxylation of citric acid [31].

Carbohydrate-derived compounds, including monosaccharides such as glucose and ribose, as well as minor degradation products such as 5-hydroxymethylfurfural (HMF), were also detected. This indicates partial hydrolysis of hemicellulosic fractions combined with limited sugar degradation, as previously reported for citric acid treatments by other authors [32].

A wide range of lignin-derived phenolic compounds was identified, including sinapic acid, coumaric acid, and 4-methylguaiacol, corresponding to S, H, and G lignin units, respectively. This indicates that partial lignin depolymerization occurred under the selected conditions without extensive oxidation, as intact phenolic monomers were preserved. Nevertheless, some phenolic compounds underwent secondary degradation reactions, leading to the formation of hydroxybenzaldehydes and 3,4-dihydroxybenzaldehyde/4-hydroxybenzoic acid, which are associated with the breakdown of H units or condensed tannins [33,34]. The presence of catechol is particularly relevant, as it may originate from the demethylation of lignin-derived G units or from the degradation of condensed tannins [35,36].

Finally, several secondary metabolites were detected, including 2,3-dihydroxypropyl 3,4,5-trihydroxybenzoate (derived from gallic acid present in hydrolyzable tannins), coumarins (such as scopoletin or isocoumarin derivatives), phytoalexins such as 2,3,4-trihydroxybenzophenone, dimeric lignans including 1-acetoxypinoresinol and (+)-lyoniresinol, and lipid-derived compounds such as azelaic acid. These results indicate that the pretreatment not only solubilized structural components of the lignocellulosic matrix but also released intracellular and extractive-derived metabolites [33].

The composition of the extracted liquor is consistent with previous studies on mild acidic and hydrothermal pretreatment of lignocellulosic residues, where complex mixtures of phenolic compounds, sugar-derived products, organic acids, and secondary metabolites are commonly obtained [32,33,35]. Compared with more severe treatments, the conditions applied in this work appear to preserve a diverse phenolic and secondary metabolite profile while limiting extensive degradation [33]. Stilbenes were not among the predominant compounds, in contrast to studies focused on organic solvent extraction of vine shoot phenolics [8,37]. This difference is probably related to their limited water solubility and to the acidic hydrothermal conditions used in the present work. Nevertheless, the detection of gallic acid-related compounds, coumarins, hydroxybenzaldehydes, catechol, and phytoalexin-related structures agrees with previous reports on vine shoot extracts and hydrothermal liquors [33,38].

In terms of lignin-derived phenolics, the profile obtained from vine shoots also resembles those reported for other lignocellulosic biomasses, such as eucalyptus, poplar wood, and rice husks, where benzaldehyde derivatives, guaiacol-type compounds, coumaric acid, and syringyl/sinapyl derivatives are commonly generated during acidic or hydrothermal processing [35,39,40].

Overall, the acidic pretreatment produced a phenolic-rich liquor containing a diverse mixture of bioactive and reactive compounds, providing a suitable substrate for subsequent enzymatic modification.

### 3.3. Enzymatic Oxidation of Phenolic Liquors

**Figure 5:**
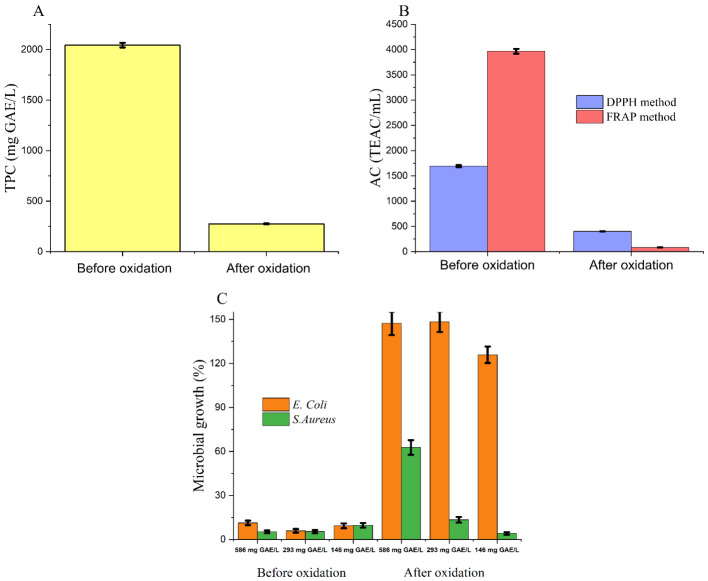
Effect of laccase treatment on the functional properties of the phenolic-rich liquor obtained under optimized pretreatment conditions. (**A**) Total phenolic content (TPC). (**B**) Antioxidant capacity determined by DPPH and FRAP assays. (**C**) Antimicrobial activity against *Escherichia coli* and *Staphylococcus aureus* at different liquor concentrations before and after enzymatic oxidation.

Before evaluating starch functionalization, the reactivity of the hemicellulose-derived phenolic liquor toward laccase-catalyzed oxidation was assessed. This experiment was designed as a preliminary reactivity study to determine whether the phenolic compounds present in the complex liquor could act as substrates for laccase-mediated transformation. For this purpose, the liquor was treated with laccase for 24 h in the absence of starch, and the resulting changes in total phenolic content, antioxidant capacity, and antimicrobial activity were analyzed (Figure 5). To further support enzymatic transformation, oxygen consumption during the initial stage of laccase treatment was monitored (Figure S1). A rapid decrease in oxygen was observed after enzyme addition, supporting laccase-mediated oxidative activity in the liquor. In addition, HPLC-DAD chromatograms before and after laccase treatment showed a general reduction in the area of several UV-absorbing peaks at 280 nm (Figure S2), consistent with the depletion of low-molecular-weight phenolic compounds.

Control experiments were carried out to distinguish laccase-catalyzed oxidation from non-enzymatic changes during incubation. Phenolic liquor incubated without enzyme or with heat-inactivated MtL under the same reaction conditions showed only minor changes, with TPC decreasing by approximately 5% and antioxidant capacity decreasing by 6.5% and 7.5% according to the DPPH and FRAP assays, respectively. These results indicate that some marginal non-enzymatic oxidation occurred under the aerated reaction conditions, as also suggested by the oxygen profile in Figure S1, but that the marked decrease observed in the presence of active laccase was mainly associated with enzymatic oxidation.

As shown in Figure 5, laccase treatment led to a marked decrease in both TPC and antioxidant capacity after 24 h of reaction. This decrease is consistent with the enzymatic oxidation of low-molecular-weight phenolic compounds and their conversion into products with lower response in the Folin–Ciocalteu, DPPH, and FRAP assays, including possible oxidized and/or coupled structures. Therefore, the loss of antioxidant capacity should not be interpreted only as a loss of functionality, but also as evidence that the liquor contains laccase-reactive phenolic substrates able to undergo oxidative transformation.

The antimicrobial properties of the untreated liquor showed strong inhibition against both Gram-negative (*Escherichia coli*) and Gram-positive (*Staphylococcus aureus*) bacteria, reducing bacterial growth to approximately 15–20%, even at low concentrations (Figure 5C). This inhibitory effect can be attributed to the presence of low-molecular-weight phenolic compounds, as well as sugar-derived degradation products such as furfural and 5-hydroxymethylfurfural (HMF), which are known to exert antimicrobial activity [10].

After enzymatic oxidation, a clear reduction in antimicrobial activity was observed. This behavior is consistent with the transformation of reactive phenolic compounds into less bioactive, higher molecular weight structures, thereby decreasing their inhibitory effect. In the case of *E. coli*, the oxidized liquor even promoted bacterial growth, suggesting that the reduction in inhibitory phenolics, combined with the presence of residual sugars and oligosaccharides, created more favorable growth conditions. Similar behavior was observed for *S. aureus*, although a certain level of inhibition was still maintained [3]. The remaining antimicrobial effect after oxidation may be attributed to compounds that are less reactive under laccase treatment conditions, such as furfural and HMF, which can contribute to bacterial inhibition, particularly in Gram-positive strains. Detoxification effects after laccase treatment have also been previously reported [41]. In that study, yeast growth in the presence of sugars and phenolic compounds improved after enzymatic oxidation, which was associated with the reduction in phenolic toxicity and enhanced glucose utilization. This behavior was attributed to the oxidative transformation of membrane-disrupting phenolics such as catechol and gallic acid, both detected in the present VS liquor [41]. Accordingly, laccase treatment has been widely investigated as a detoxification strategy for biomass-derived phenolic liquors [42]. For example, laccase was employed for the detoxification of wheat straw steam exploded liquor, enhancing the sugar consumption for bioethanol production [43].

In the present work, however, this oxidation step was not only considered as a detoxification process. Its main purpose was to demonstrate that the phenolic compounds in the vine shoot liquor were reactive toward laccase and could therefore be activated for subsequent coupling reactions. This behavior is particularly relevant for complex biomass-derived streams, where the reactivity of phenolic mixtures remains less explored compared to purified model compounds. Previous studies have explored the possibilities of treating a complex phenolic liquor from wine lees with laccase to produce polymers [24,43]. For example, Jurado et al. reported the production of a phenolic polymer from steam-exploded liquor after detoxification with *Candida rigida* laccase [43].

These results show that laccase treatment substantially modified the phenolic-rich liquor, decreasing its Folin-reactive phenolic content, antioxidant capacity, and antimicrobial activity. Together with the oxygen consumption profile and HPLC-DAD chromatograms, these data support the enzymatic transformation of low-molecular-weight phenolic compounds present in the liquor. Although the specific oxidation and coupling products were not fully identified, the observed changes indicate that the liquor contains laccase-reactive phenolic substrates suitable for subsequent functionalization experiments with starch.

### 3.4. Laccase-Mediated Starch Functionalization

**Figure 6:**
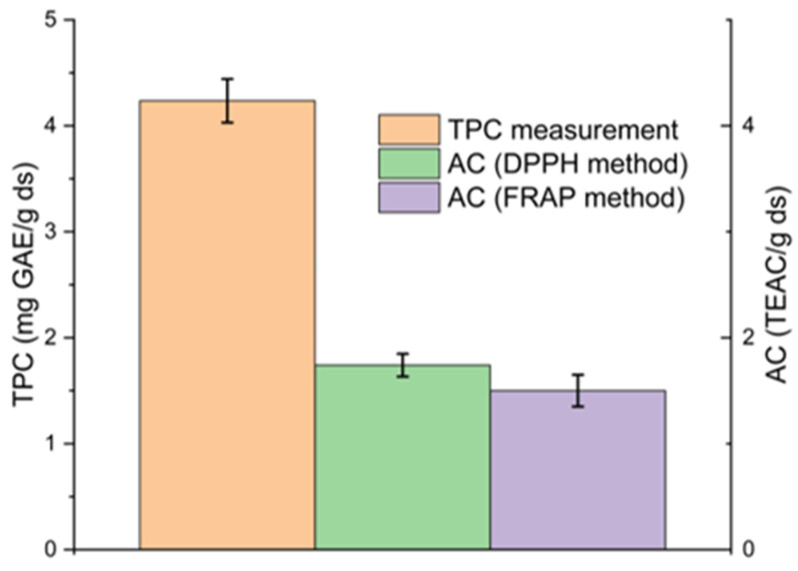
Phenolic content and antioxidant capacity of starch after laccase-mediated treatment in the presence of vine shoot-derived phenolic-rich liquor. Native starch was used as a control.

Laccase-catalyzed oxidation of the hemicellulose-derived phenolic liquor was carried out in the presence of starch to evaluate an in situ enzymatic functionalization approach. In this configuration, starch was present during the enzymatic oxidation step and therefore acted as a potential acceptor matrix for reactive phenolic intermediates generated by laccase. The formation of phenoxy radicals during laccase treatment can promote coupling reactions among oxidized phenolics and may also favor their association or incorporation into surface-accessible regions of the starch matrix. After reaction, the resulting solid was extensively washed to remove non-retained compounds and subsequently analyzed in terms of TPC and antioxidant capacity. The results are presented in Figure 6.

As shown in Figure 6, the modified starch exhibited a phenolic content above 4 mg GAE g^−1^ starch, whereas native starch showed a negligible response under the same assay conditions.

Control experiments were carried out to distinguish laccase-mediated phenolic incorporation from passive adsorption or non-enzymatic oxidation. The control experiments are particularly relevant because starch incubated with phenolic liquor in the absence of active enzyme retained only 0.86 ± 0.05 mg GAE g^−1^ starch. This low value indicates that passive adsorption of Folin-reactive compounds onto starch was limited under the reaction conditions. Nevertheless, this retention cannot be attributed exclusively to adsorption, since the oxygen consumption profile of the liquor without enzyme indicated that limited autoxidation also occurred during incubation (Figure S1). Therefore, the low TPC detected in this control may arise from a combination of weak adsorption and non-enzymatic oxidation/association of phenolic compounds. Importantly, no measurable antioxidant capacity was detected in this control, indicating that the retained Folin-reactive material did not confer detectable redox functionality to the starch. The heat-inactivated laccase control retained a slightly higher TPC value, 1.35 ± 0.11 mg GAE g^−1^ starch. Indeed, the heat-inactivated laccase retained less than 10% of the initial activity according to the catechol oxygen-consumption assay, indicating that the deactivation step substantially reduced, but did not completely abolish, enzymatic activity. However, no measurable antioxidant capacity was detected in this control either. Importantly, both controls showed substantially lower phenolic retention and no detectable antioxidant capacity compared with the active-laccase treatment, supporting the contribution of laccase-mediated oxidation to phenolic incorporation into a functionally active washed starch fraction.

The retention of phenolic compounds after repeated washing indicates that liquor-derived phenolics were strongly incorporated into or associated with the starch matrix during enzymatic treatment. Although additional structural characterization would be required to illustrate covalent grafting, the results support the occurrence of an in situ enzymatic functionalization process. Analysis of the reaction supernatant after starch separation showed only minor changes compared with the oxidized liquor obtained in the absence of starch. This result is consistent with the mass balance of the system: the initial reaction contained approximately 20 mg GAE, whereas the washed starch retained approximately 1 mg GAE, corresponding to about 5% of the initial Folin-reactive content. Therefore, the corresponding decrease in the supernatant is expected to be small and difficult to resolve within the variability of the Folin–Ciocalteu assay, particularly given the complex composition of the liquor.

In addition to phenolic incorporation, the functionalized starch retained measurable antioxidant capacity, reflecting the presence of retained Folin-reactive structures with redox activity after enzymatic treatment. Although laccase oxidation reduced the antioxidant capacity of the free liquor (Figure 5), part of the phenolic functionality was retained in the washed starch fraction when starch was present during the oxidative process. Thus, enzymatic oxidation appears to act not only as a transformation step for the liquor, but also as an activation strategy favoring phenolic incorporation into a polysaccharide matrix.

Previous studies have reported higher phenolic incorporation when using purified phenolic compounds or polymers with more reactive functional groups. For example, starch-quercetin conjugates prepared by chemical radical grafting reached phenolic contents around 13 mg g^−1^ starch [44]. Similarly, laccase-mediated functionalization of pectin with ferulic acid-derived oxidation products achieved higher phenolic incorporation, which can be related to the water solubility of pectin and its native association with phenolic ester linkages [45]. Chitosan has also been widely used for phenolic grafting because its amine groups favor coupling reactions, with reported values around 37 mg GAE g^−1^ DS when ferulic acid was incorporated into carboxymethyl chitosan [46]. In comparison, the lower phenolic content obtained in the present work is expected because starch offers fewer highly reactive functional groups and because the phenolic source was not a purified model compound, but a complex vine-shoot-derived liquor.

This distinction is relevant from a valorization perspective. In contrast to conventional strategies based on isolated phenolic compounds, the present approach directly uses a complex liquor obtained from biomass pretreatment. This reduces the need for extensive purification and enables the coupling of extraction and enzymatic upgrading in a single valorization route. Only a limited number of studies have explored the use of biorefinery-derived liquors or lignin-rich streams for direct polymer modification. For instance, oxidized kraft lignin has been reacted in the presence of polyethyleneimine, chitosan, or soy protein to produce grafted polymers with improved adhesive properties [47]. However, the direct use of vine-shoot-derived phenolic liquors for starch functionalization is reported here for the first time.

Since direct structural evidence of specific covalent bonds between liquor-derived phenolics and starch was not obtained, the term grafting, when used, is used only in an operational sense to describe laccase-mediated phenolic incorporation/retention in the washed starch fraction. Further structural analyses, such as solid-state NMR, FTIR, or MS-based analysis of extracted fractions, will be required to unequivocally establish covalent bond formation.

Overall, this approach demonstrates the potential of enzymatically modified phenolic liquors for the functionalization of polysaccharide-based materials, providing a route to obtain bio-based materials with enhanced antioxidant properties. Future work should optimize the enzymatic activation step by evaluating enzyme loading, pH, temperature, reaction time, and oxygen availability, ideally using response surface methodology to balance phenolic activation, antioxidant retention, and incorporation efficiency into polysaccharide matrices.

The modified starch obtained in this work should be considered a functionalized starch intermediate rather than a fully developed packaging material. Therefore, material-level properties such as film-forming behavior, mechanical performance, crystallinity, swelling, migration, and thermal stability were beyond the scope of this proof-of-concept study.

## 4. Conclusions

Vine shoot residues were successfully valorized through citric acid-assisted pretreatment, producing phenolic-rich liquors containing lignin-derived compounds, carbohydrate derivatives, organic acids, and secondary metabolites. Temperature was the main factor controlling both hemicellulose solubilization and phenolic release, and the optimized conditions enabled the recovery of liquors with high total phenolic content.

Citric acid-assisted pretreatment of vine shoots was optimized at 190 °C, 75 min, and 0.82% citric acid, reducing residual hemicellulose in the pretreated solid to 2.9 ± 0.02% and increasing cellulose and lignin contents to 47.5 ± 0.20% and 51.8 ± 1.87%, respectively. Under these conditions, the process generated a phenolic-rich liquor with 27.66 ± 0.39 mg GAE g^−1^ dry solid and a complex profile of lignin-derived phenolics, carbohydrate derivatives, organic acids, and secondary metabolites. Laccase treatment at pH 7.0 and 30 °C reduced the Folin-reactive phenolic content and antioxidant capacity of the liquor, while also decreasing its antimicrobial activity, indicating enzymatic transformation of reactive phenolic compounds. When starch was present during laccase oxidation, the washed solid retained approximately 4 mg GAE g^−1^ starch and measurable antioxidant capacity, supporting laccase-mediated phenolic incorporation into the starch matrix.

Overall, this study demonstrates the feasibility of coupling vine shoot fractionation with enzymatic upgrading of phenolic-rich liquors to obtain functionalized starch intermediates. Nevertheless, several limitations should be considered. First, the present results do not unequivocally establish specific covalent linkages between phenolics and starch, and the Folin–Ciocalteu assay should be interpreted as a measure of Folin-reactive retained compounds rather than as direct proof of grafting. Second, the enzymatic activation step was evaluated under a single set of reaction conditions and should be optimized in future work with respect to enzyme loading, pH, temperature, reaction time, oxygen availability, and the phenolic-to-matrix ratio. Systematic variation in these parameters would provide a basis for modulating and controlling phenolic loading on starch and other polysaccharide matrices, allowing the functionalization degree to be tailored to the intended application. Finally, industrial implementation would require addressing raw material logistics, seasonal availability of vine shoots, enzyme cost, enzyme reuse or immobilization, liquor purification requirements, and the performance of the modified starch in specific food-related applications. Future research should therefore focus on structural confirmation of phenolic incorporation, leaching and migration assays, immobilized laccase systems, process integration, material-level characterization, and the extension of this approach to composite polysaccharide matrices.

## Figures and Tables

**Table 1:** Experimental design for hemicellulose extraction by Box–Behnken design and the different responses measured. H: hemicellulose content; C: cellulose content; L: lignin content.

Exp	T (°C)	t (Min)	% Citric Acid (p/v)	H (%)	C (%)	L (%)
1	130	30	0.5	18.3	37.2	36.9
2	190	30	0.5	3.9	46.2	49.4
3	130	90	0.5	18.1	37.9	37.7
4	190	90	0.5	2.2	44.7	52.4
5	130	60	0	18.8	37.7	36.7
6	190	60	0	3.9	46.5	49.3
7	130	60	1	19.5	38.3	36.3
8	190	60	1	2.4	48.8	48.4
9	160	30	0	17.5	37.8	41.2
10	160	90	0	14.3	42.8	40.1
11	160	30	1	11.0	42.3	44.0
12	160	90	1	8.12	44.9	45.1
13	160	60	0.5	9.9	44.7	44.1
14	160	60	0.5	10.2	44.3	45.2
15	160	60	0.5	10.0	44.5	42.8

**Table 2:** Major metabolites tentatively annotated via UHPLC-timsTOF MS in the liquor obtained under optimal conditions.

Compound Number	Rt (min)	*m*/*z* Meas.	Molecular Formula	Error (ppm)	mSigma	CCS (Å^2^)	% Relative Intensity	Tentative Identity
1	2.78	191.0197	C_6_H_8_O_7_	0.03	3.2	123.4	100	Citric acid
2	8.32	191.0349	C_10_H_8_O_4_	−0.313	5.3	134.1	40.5	Scopoletin
3	6.98	109.0293	C_6_H_6_O_2_	−1.572	3.4	110.1	37.4	Pyrocatechol
4	3.17	147.0299	C_5_H_8_O_5_	−0.285	0.6	118	31.1	Citramalic acid
5	6.5	137.0242	C_7_H_6_O_3_	−1.647	3.4	114.9	29.3	3,4-Dihydroxy-benzaldehyde/ 4-Hydroxybenzoic acid
6	3.59	129.0192	C_5_H_6_O_4_	−0.763	10.2	113.5	18.5	Citraconic acid
7	11.63	229.0507	C_13_H_10_O_4_	0.344	11.7	147.3	15.1	2,3,4-Trihydroxibenzophenone
8	2.19	149.0455	C_5_H_10_O_5_	−0.591	9	120.4	12.7	Xylose
9	8.14	181.0504	C_7_H_6_O_2_	−1.813	6.3	128.9	12.4	Hydroxybenzaldehyde
10	2.07	179.0562	C_6_H_12_O_6_	0.293	8.3	127.7	12.3	α-D-glucose
11	9.62	415.1396	C_22_H_24_O_8_	−0.656	11.1	193.7	10.6	1-Acetoxypinoresinol
12	8.52	419.1713	C_22_H_28_O_8_	0.24	3.8	210.3	8.3	(+)-Lyoniresinol
13	11.64	223.0613	C_11_H_12_O_5_	0.431	2.5	144.5	8.1	Sinapic acid
14	4.59	243.0512	C_10_H_12_O_7_	0.712	11.9	145.2	7.9	2,3-Dihydroxypropyl 3,4,5-trihydroxybenzoate
15	3.35	181.0143	C_8_H_6_O_5_	0.453	4.9	123.9	7.3	2-(3,4-Dihydroxyphenyl)-2-oxoacetic acid
16	10.03	187.0975	C_9_H_16_O_4_	−0.537	1.1	136.1	7.1	Azelaic acid
17	6.42	163.0399	C_9_H_8_O_3_	−1.27	1.3	126	7.0	Coumaric acid
18	8.67	235.0248	C_11_H_8_O_6_	0.081	14.5	142.3	7.0	6,7-Dihydroxy-4-coumarinylacetic acid
19	8.14	137.0607	C_8_H_10_O_2_	−1.019	5.5	121.3	7.0	4-Methylguaiacol
20	8.85	121.0293	C_7_H_6_O_2_	−1.366	5.1	113.6	5.9	3-Hydroxybenzaldehyde

Note: *m*/*z* values correspond to [M − H]^−^, except for compound 9, which was detected as the acetate adduct [M + CH_3_COO]^−^, and compound 16, which was detected as the water loss [M − H − H_2_O]^−^. Error (ppm): Mass accuracy; mSigma: Isotopic pattern match score (lower values indicate higher confidence); CCS (Å^2^): Experimental collision cross section value; % Relative Intensity: Peak intensity expressed as a percentage relative to the base peak (most intense ion, 100%); Tentative Identity: Annotation based on accurate mass, MS/MS fragmentation, CCS values, and literature comparison.

## Data Availability

The original contributions presented in this study are included in the article/Supplementary Materials. Further inquiries can be directed to the corresponding author.

## Supplementary Materials

The following supporting information can be downloaded at: https://www.mdpi.com/article/10.3390/foods15122177/s1, Figure S1: Oxygen consumption during the initial oxidation of phenolic liquor by MtL in the presence of starch, complete oxidation of phenolic liquor by MtL alone, and phenolic liquor autoxidation without MtL addition; Figure S2: HPLC-DAD chromatograms of the phenolic liquor before and after oxidation by MtL, recorded at 280 nm; Figure S3: Visual appearance of starch samples after incubation under the different treatment conditions: native starch incubated with MtL, starch incubated with phenolic liquor alone, starch incubated with phenolic liquor and heat-inactivated MtL, and starch incubated with MtL and phenolic liquor; Table S1: Additional tentatively annotated metabolites identified by UHPLC-timsTOF-MS in the liquor obtained under optimal conditions.

## Abbreviations

The following abbreviations are used in this manuscript:

AC	Antioxidant capacity
ANOVA	Analysis of variance
BBD	Box–Behnken design
C	Cellulose
CCS	Collision cross section
CECT	Spanish Type Culture Collection
DES	Deep eutectic solvents
DPPH	2,2-diphenyl-1-picrylhydrazyl
FRAP	Ferric reducing antioxidant power
g ds	Grams of dry solid
GAE	Gallic acid equivalents
H	Hemicellulose
HMF	5-hydroxymethylfurfural
HPLC-DAD	High-performance liquid chromatography–diode-array detector
L	Lignin
L/S	Liquid-to-solid ratio
NREL	National Renewable Energy Laboratory
RID	Refractive index detector
RSM	Response surface methodology
TEAC	Trolox equivalent antioxidant capacity
TIMS	Trapped ion mobility spectrometry
TPC	Total Phenolic Content
TPTZ	2,4,6-tri(2-pyridyl)-s-triazine
UHPLC-timsTOF-MS	Ultra-high-performance liquid chromatography coupled to trapped ion mobility time-of-flight mass spectrometry
VSs	Vine shoots
