# Supplementary material for: Valorization of Vine Shoot Waste into Phenolic-Rich Liquors for Laccase-Mediated Functionalization of Starch
Source: Foods. 2026 Jun 16;15(12):2177. doi: 10.3390/foods15122177 (PMC13297696; doi:10.3390/foods15122177)
Supplement: Supplementary file 1 [file foods-15-02177-s001.zip › foods-4353288-supplementary.pdf]

Supplementary Materials

Valorization of Vine Shoot Waste into Phenolic-Rich Liquors for Laccase-Mediated Functionalization of Starch

García-Montalvo, Jorge[1], Olmo-García, Lucía[2], Moreno-Rúa, Nuria[1], Oreja-Remartínez, David[1], Fernández-Sánchez, Jorge[2], Carrasco-Pancorbo, Alegría[2], Ladero, Miguel[1], Bolivar, Juan M.[1]*

[1] FQPIMA group, Chemical and Materials Engineering Department, Faculty of Chemical Sciences, Complutense University of Madrid, Madrid, 28040, Spain

[2] Department of Analytical Chemistry, Faculty of Sciences, University of Granada, Ave. Fuentenueva s/n, 18071 Granada, Spain

* Correspondence: juanmbol@ucm.es.

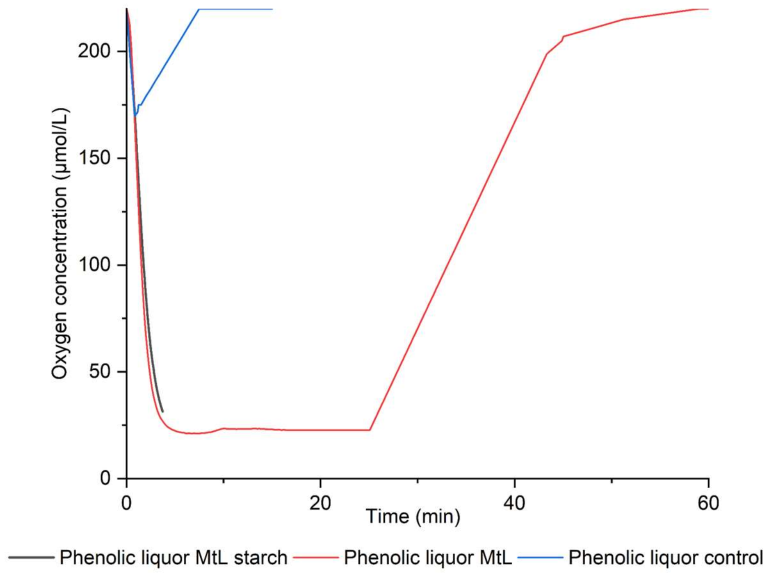

Figure S1. Oxygen consumption during the initial oxidation of phenolic liquor by MtL in the presence of starch, oxidation of phenolic liquor by MtL alone, and phenolic liquor autoxidation in the absence of MtL.

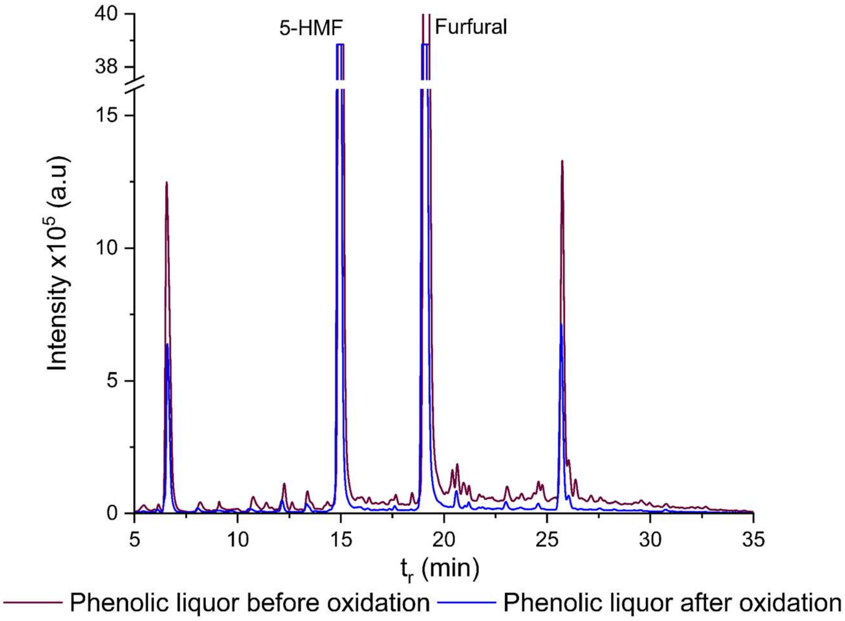

Figure S2: HPLC-DAD chromatograms of the phenolic liquor before and after oxidation by MtL recorded at 280 nm.

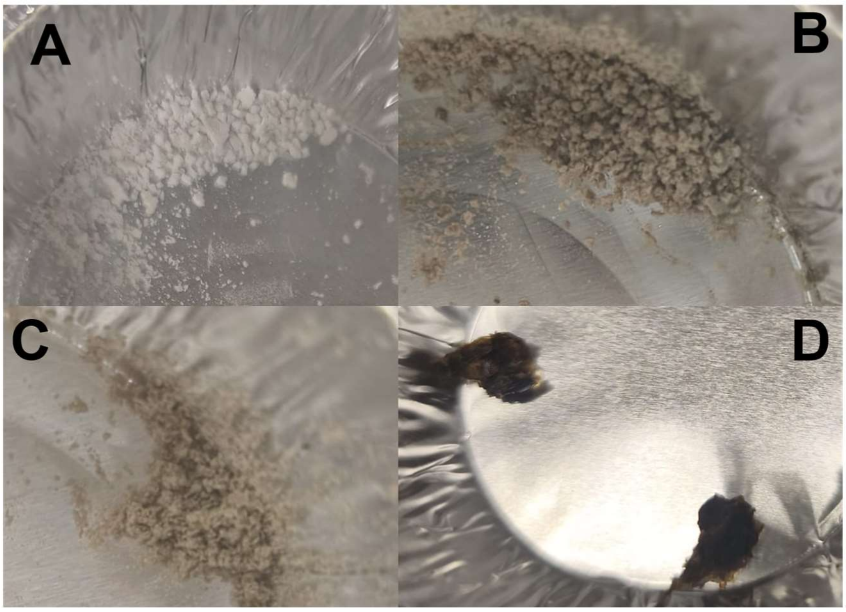

Figure S3: Visual appearance of starch samples after incubation under the different treatment conditions: (A) native starch incubated with MtL, (B) starch incubated with phenolic liquor alone, used as adsorption control, (C) starch incubated with phenolic liquor and heat-inactivated MtL under reaction conditions, and (D) starch incubated with MtL and phenolic liquor.

Table S1: Additional tentatively annotated metabolites (21–81 by relative signal intensity) identified by UHPLC-timsTOF-MS in the liquor obtained under optimal conditions.

Compound number	Rt (min)	*m/z* meas.	Molecular Formula	Error (ppm)	mSigma	CCS (Å²)	% Relative Intensity	Tentative Identity
21	11.57	193.0504	$C_{10}H_{10}O_4$	-1.096	8.5	135.2	5.9	Ferulic acid
22	6.19	225.0768	$C_{11}H_{14}O_5$	-0.147	10.2	141.4	5.8	3 4 5-trimethoxyphenylacetic
23	4.76	243.0508	$C_{10}H_{12}O_7$	-0.794	11.6	143.2	5.7	2,3-Dihydroxypropyl 3,4,5-trihydroxybenzoate
24	4.21	173.0454	$C_7H_{10}O_5$	-0.977	7.8	127.3	5.4	Shikimic acid
25	4.79	153.0192	$C_7H_6O_4$	-0.794	9.7	117.6	5.0	Protocatechuic acid
26	3.34	109.0293	$C_6H_6O_2$	-2.045	4.3	110.0	4.8	Hydroquinone
27	8.89	181.0504	$C_9H_{10}O_4$	-1.077	4.4	131.3	4.8	Veratric acid
28	2.78	111.0087	$C_5H_6O_4$	-2.116	11.7	105.7	4.6	Citraconic acid isomer
29	4.43	125.0243	$C_6H_6O_3$	-0.863	4.3	112.2	4.5	Hydroxymethylfurfural
30	9.38	135.0450	$C_8H_8O_2$	-1.226	5.4	120.8	4.5	4-Hydroxyacetophenone
31	4.9	243.0508	$C_{10}H_{12}O_7$	-0.790	4.0	143.0	4.3	2,3-Dihydroxypropyl 3,4,5-trihydroxybenzoate
32	3.04	131.0348	$C_5H_8O_4$	-1.566	5.6	117.9	4.2	Glutaric acid
33	4.95	193.0506	$C_{10}H_{10}O_4$	-0.006	2.4	134.6	4.2	Ferulic acid isomer
34	2.5	637.1843	$C_{22}H_{38}O_{21}$	2.548	13.8	222.8	4.2	Maltotriosyl-quercitol
35	7.97	269.0667	$C_{12}H_{14}O_7$	0.199	2.9	147.9	4.1	Phenyl glucuronide
36	8.61	259.0612	$C_{14}H_{12}O_5$	-0.049	13.6	153.6	4.0	3,4,5,2′,4′-Pentahydroxystyrene (resveratrol derivative)
37	4.86	253.0354	$C_{11}H_{12}O_8$	0.846	14.9	145.4	4.0	3,4- Dihydroxybenzyltartaric acid
38	11.63	229.0505	$C_{13}H_{10}O_4$	-0.645	0.4	213.4	4.0	2,4,6-Trihydroxybenzophenone
39	7.83	181.0505	$C_9H_{10}O_4$	-0.670	7.5	129.5	3.9	Homovainillic acid
40	8.73	151.0399	$C_8H_8O_3$	-1.329	6.2	122.2	3.9	Vanillin
41	4.96	225.0403	$C_{10}H_{10}O_6$	-0.538	10.4	137.7	3.9	4-(2-Carboxy-2-hydroxyethyl)-3-hydroxybenzoic acid
42	5.66	209.0455	$C_{10}H_{10}O_5$	-0.157	10.0	137.1	3.8	2-(2-Carboxyethyl)-5-hydroxybenzoic acid
43	9.74	305.1031	$C_{16}H_{18}O_6$	0.439	7.3	170.3	3.7	6,8-Dimethoxy-3-methyl-3,4-dihydrobenzoisochromene-4,9,10-triol
44	6.76	205.0507	$C_{11}H_{10}O_4$	0.511	8.7	142.1	3.7	Scoparone
45	10.25	207.0662	$C_{11}H_{12}O_4$	-0.576	9.0	141.5	3.7	3,4-Dimethoxycinnamic acid
46	7.07	151.0399	$C_8H_8O_3$	-1.359	6.8	121.6	3.6	Vanillin isomer

47	3.31	137.0243	$C_7H_6O_3$	-0.853	5.2	114.8	3.6	4-Hydroxybenzoic acid
48	9.43	287.1500	$C_{14}H_{24}O_6$	-0.161	15.1	160.0	3.5	(E)-9,10-Dihydroxytetradec-6-enedioic acid
49	5.6	269.0667	$C_{12}H_{14}O_7$	0.083	14.1	148.5	3.5	Phenyl glucuronide
50	6.81	201.0768	$C_9H_{14}O_5$	-0.416	9.6	136.2	3.4	Oxoazealic acid
51	3.86	261.0615	$C_{10}H_{14}O_8$	-0.550	3.5	147.0	3.4	Diacetyl galactaric acid
52	9.93	123.0450	$C_7H_8O_2$	-1.035	4.9	115.9	3.3	4-Methyl catechol
53	9.87	275.0926	$C_{15}H_{16}O_5$	0.111	14.2	161.5	3.3	3,3-Diphenylpropane-1,1,2,2,3-pentol
54	10.84	243.1237	$C_{12}H_{20}O_5$	-0.166	10.8	149.9	3.3	4-Oxododecanedioic
55	3.21	259.0454	$C_{10}H_{12}O_8$	-2.118	13.5	149.4	3.2	Galloyl-glycerol derivative (Oxidized form)
56	11.71	417.1555	$C_{22}H_{26}O_8$	0.018	5.2	209.7	3.1	Syringaresinol
57	7.46	221.0455	$C_{11}H_{10}O_5$	0.618	9.9	141.5	3.1	Fraxinol
58	4.73	167.0349	$C_8H_8O_4$	-0.673	7.3	123.2	3.0	Vanillic acid
59	6.59	247.0610	$C_{13}H_{12}O_5$	-0.555	11.8	150.5	3.0	8-Acetyl-6,7-dimethoxycoumarin
60	10.39	177.0556	$C_{10}H_{12}O_4$	-0.501	7.3	132.2	3.0	Acetosyringone
61	5.82	129.0556	$C_6H_{10}O_3$	-0.785	5.7	121.9	2.9	4-Oxohexanoic acid
62	5.29	153.0193	$C_7H_6O_4$	-0.411	6.3	118.3	2.9	Protocatechuic acid
63	4.7	131.0348	$C_5H_8O_4$	-1.120	7.9	116.6	2.8	Glutaric acid
64	13.54	213.0557	$C_{13}H_{10}O_3$	-0.036	7.4	144.9	2.8	4,4'-Dihydroxybenzophenone
65	7.71	247.0251	$C_{12}H_8O_6$	0.976	0.3	141.9	2.7	7-Deshydroxypyrogallin-4-carboxylic acid
66	9.12	359.1500	$C_{20}H_{24}O_6$	0.138	1.6	185.7	2.2	4,3',5'-Tris(2-hydroxyethoxy)stilbene (resveratrol derivative)
67	11.07	387.1448	$C_{21}H_{24}O_7$	-0.336	7.4	190.6	2.0	Medioresinol
68	5.09	227.0561	$C_{10}H_{12}O_6$	0.031	11.2	142.6	2.0	Carlosic acid
69	12.16	249.0769	$C_{13}H_{14}O_5$	0.120	0.4	148.5	1.9	Methyl 4-acetoxy-3-methoxycinnamate
70	5	239.0558	$C_{11}H_{12}O_6$	-1.426	12.5	143.5	1.8	Acetyl syringic acid
71	4.09	273.0615	$C_{11}H_{14}O_8$	-0.323	21.7	152.0	1.8	Tri-O-acetyl-xylonic acid
72	9.02	403.1397	$C_{21}H_{24}O_8$	-0.512	3.4	193.9	1.7	Fraxiresinol
73	9.18	261.1344	$C_{12}H_{22}O_6$	0.054	15.4	155.3	1.6	trans/cis 2-hexen-1-ol b-D-glucopyranoside
74	5.19	277.0716	$C_{14}H_{14}O_6$	-0.561	13.6	157.9	1.5	4-[2-(3,5-Dihydroxyphenyl)-1,2-dihydroxyethyl]benzene-1,2-diol
75	7.63	253.0717	$C_{12}H_{14}O_6$	-0.150	12.8	147.2	1.5	Methyl 4-acetyloxy-3,5-dimethoxybenzoate
76	7.29	179.0349	$C_9H_8O_4$	-0.483	7.3	137.8	1.2	Caffeic acid
77	11.23	341.1030	$C_{19}H_{20}O_7$	-0.108	20.9	183.2	1.1	2,5-Dimethoxyphenyl 3-acetoxy-2-hydroxy-3-phenylpropanoate (resveratrol derivative)
78	11.44	305.1030	$C_{16}H_{18}O_6$	-0.265	7.5	166.7	1.1	6,8-Dimethoxy-3-methyl-3,4-dihydrobenzoisochromene-4,9,10-triol
79	10.74	403.1395	$C_{21}H_{24}O_8$	-0.834	5.3	204.8	1.0	trans-Pinostilbene-4'-O-glucoside
80	5.9	257.0666	$C_{11}H_{14}O_7$	-0.323	14.2	150.6	1.0	Phloroglucinol xyloside
81	10.55	403.1401	$C_{21}H_{24}O_8$	0.593	36.6	239.8	0.9	trans-Pinostilbene-4'-O-glucoside

Note: *m/z* values correspond to $[M-H]^-$, except for compounds 28, 37, 60, and 77, which were detected as the water loss $[M-H-H_2O]^-$. **Error (ppm):** Mass accuracy; **mSigma:** Isotopic pattern match score (lower values indicate higher confidence); **CCS (Å²):** Experimental collision cross section value; **% Relative Intensity:** Peak intensity expressed as a percentage relative to the base peak (most intense ion, 100%); **Tentative Identity:** Annotation based on accurate mass, MS/MS fragmentation, CCS values, and literature comparison.
